# Circulating Mitochondrial DAMPs Are Not Effective Inducers of Proteinuria and Kidney Injury in Rodents

**DOI:** 10.1371/journal.pone.0124469

**Published:** 2015-04-22

**Authors:** Jing He, Yuqiu Lu, Hong Xia, Yaojun Liang, Xiao Wang, Wenduona Bao, Shifeng Yun, Yuting Ye, Chunxia Zheng, Zhihong Liu, Shaolin Shi

**Affiliations:** 1 National Clinical Research Center of Kidney Diseases, Jinling Hospital, Nanjing University School of Medicine, Nanjing, China; 2 Department of Comparative Medicine, Jinling Hospital, Nanjing University School of Medicine, Nanjing, China; Charité Universitätsmedizin Berlin, GERMANY

## Abstract

Mitochondria in eukaryotic cells are derived from bacteria in evolution. Like bacteria, mitochondria contain DNA with unmethylated CpG motifs and formyl peptides, both of which have recently been shown to be damage associated molecular patterns (DAMPs) and induce immune response and cell injury. Based on the facts that circulating mitochondrial DAMPs (mtDAMPs) are increased in the patients of trauma or burn injury who also have proteinuria, that mtDAMPs can activate immune cells which in turn secrete glomerular permeability factors, that renal intrinsic cells express a variety of DAMP receptors, and that mtDAMPs can directly increase endothelial cell permeability *in vitro*, we hypothesized that mtDAMPs may be novel circulating factors inducing proteinuria and kidney injury. We tested this hypothesis by directly injecting mtDAMPs into rodents and examining urinary protein and kidney histology. We prepared mtDAMP samples, including mitochondrial DNA (mtDNA) and mitochondrial debris (MTD), from rodent liver. In mice, injection of mtDNA for 20 μg/ml initial concentration in circulation (much higher than the clinical range), did not cause any renal manifestations. However, an increased dose leading to 45 μg/ml initial concentration in circulation resulted in a transient, slight increase in urinary albumin. In rats, MTD injection resulting in 450 μg/ml initial concentration of MTD protein in circulation, which was much higher than the clinical range, caused mild, transient proteinuria and lung lesions. Multiple injections of such large amount of either mtDNA or MTD into rodents on 3 consecutive days also failed in inducing proteinuria and kidney injury. In summary, clinical levels of circulating mtDAMPs do not induce proteinuria and clinically irrelevant high levels of mtDAMPs cause only a transient and slight increase in urinary protein in rodents, suggesting that circulating mtDAMPs may not be responsible for the proteinuria and kidney injury in patients with trauma, burn injury, and other diseases.

## Introduction

Pathogen associated molecular patterns (PAMPs) are the molecules that are derived from pathogens and recognized by immune cells through specific receptors, termed pattern recognition receptors (PRRs), resulting in immune responses. For instances, lipopolysaccharide (LPS) of bacteria is a PAMP and is recognized by Toll-like receptor 4 (TLR4) on cell surface; bacterial DNA that contains unmethylated CpG motifs is also a PAMP and recognized by TLR9 in the cells. Formyl modification is a characteristic of bacterial proteins, and the formyl peptides are PAMPs and can induce immune response via formyl peptide receptors (FPRs) in immune cells. In an organism, some endogenous molecules can also act on PRRs and induce immune response and sterile inflammation. These endogenous molecules are termed damaged associated molecular patterns (DAMPs). DAMPs are involved in the development of many diseases including kidney diseases. Several DAMPs, including HMGB1 (high mobility group box-1) [1], S100 protein family [2], heat shock proteins [3], uric acid [4] and certain degraded extracellular matrix molecules [5], have been implicated in renal injury. More recently, certain components of mitochondria have also been shown to act as DAMPs.

Mitochondria are the organelles producing ATP for the cells. They are considered to have evolved from ancient bacteria, thus possessing many features of bacteria, including unmethylation of CpG motifs in their DNA (mtDNA) and formyl modification of the proteins that are synthesized in mitochondria themselves. Therefore, these mitochondrial components may be DAMPs and functionally similar to bacterial PAMPs in term of immunostimulatory activity. Indeed, mtDNA was found to induce inflammation and arthritis when injected into the joints of mice [6]. Intraperitoneal injection of mitochondrial fraction, but not the fractions of cytoplasma, nuclei and cell membranes, induced inflammation in the abdomen in mice [7]. Zhang et al injected mitochondrial debris (MTD) into the circulation of rats and observed systemic inflammation response syndrome (SIRS) with increased cytokine production and acute lung injury; they further clearly demonstrated that mitochondrial DNA and formyl peptides act in the same manner as DAMPs [8].

In theory, mitochondrial DAMPs (mtDAMPs) could induce kidney injury and proteinuria for several reasons. First, mtDAMPs have been shown to stimulate immune cells to produce cytokines, and some cytokines are capable of damaging renal intrinsic cells leading to kidney injury and proteinuria. For example, cytokines can induce glomerular endothelial cell injury, leading to increased glomerular permeability and proteinuria [9, 10]. Cytokines can also injure podocytes and tubular cells through their receptors expressed on these cells, resulting in proteinuria [11, 12]. Second, mtDAMPs may directly act on renal intrinsic cells, including glomerular endothelial cells, podocytes, and tubular cells, because they express abundant DAMPs receptors [1–3, 12–16], potentially resulting in kidney injury and proteinuria. A recent finding that mtDAMPs can increase endothelial permeability through neutrophil dependent and independent pathways [17] further suggests the potential injurious effect of mtDAMPs on kidney.

Patients with severe trauma or burn injury develop systemic inflammation response syndrome (SIRS), and manifest with proteinuria [18–21]. Recently, studies have shown that circulating mtDNA levels are elevated in trauma and burn patients [22–26], as well as rodent trauma models [27]. Lam et al showed that the patients with severe trauma had 1.5x10^7^ - 3.4x10^8^ copies/ml of mtDNA (~ 0.25–5 ng/ml) in circulation, a concentration that was > 10 fold higher than that of healthy controls [22]. Gu et al showed similar plasma mtDNA levels in trauma patients [24]. Chou et al also observed similarly elevated plasma mtDNA levels in the patients with burn injury [25]. Preeclampsia patients who are characterized with placenta necrosis, hypertension and proteinuria exhibit an increase in circulating DNA [28], presumably accompanied by an increase in circulating mtDAMPs, also linking circulating mtDAMPs with proteinuria. Consistently, Goulopoulou et al found that injection of mitochondria into pregnant rats results in hypertension [29], suggesting a role of circulating mtDAMPs in the pathogenesis of preeclampsia.

Based on these studies, we hypothesized that mtDAMPs that are released from injured or dead cells into circulation may exert pathogenic effects on kidney and be the circulating factors contributing to the proteinuria in the patients with trauma or burn injury. Clarifying this issue is important because it would not only provide insights into the mechanism underlying proteinuria in these patients but also identify novel circulating factors that are responsible for renal injury in certain types of kidney diseases, eg., focal segmental glomerulosclerosis (FSGS), if the mtDAMPs in circulation would be elevated in the patients. In the present study, we performed direct injection of mtDAMPs into rodents and determined whether circulating mtDAMPs are capable of inducing proteinuria and kidney injury.

## Materials and Methods

### Animals

Specific pathogens free Balb/c mice and Sprague Dawley (SD) rats were purchased from the Research Institute of Model Organisms at Nanjing University, China. These animals were used with the approved protocol by the Animal Welfare and Care Committee at Jinling Hospital, Nanjing, China.

### Isolation of mitochondria from liver

6–8 week old Balb/c mice or 200–250 g SD rats were starved for 24 h and sacrificed. Standard method was used to isolate and purify mitochondria from rat liver [30].

### MtDNA preparation

Intact mtDNA was prepared from the purified mitochondria following the method described [30]. To prepare mtDNA fragment with a proven immune activity [6], which is located between 7767–8187 nt of the mtDNA with a length of 421 bp. PCR was performed using the primers, 5’-TAGAAACCGTCTGAACTATC-3’(forward) and 5’-CCACAGATTTCAGAGCATT-3’ (reverse). In order to obtain sufficient amount of full-length mtDNA for injection in mice, we performed PCR to amplify 9 segments of mtDNA, which together cover the full-length of mtDNA. These 9 products were mixed with equal ratios and injected into mice. The primer sequences are as follow (5’ to 3’): mt1: TATAGCTTAAGACACCTTGCCTA (forward) and CACTATTTTGCCACATAGACGAG (reverse); mt2: CCAATAAAGAAAGCGTTCAAGCTC (forward) and GGCCTTTTCGTAGTTGTATATACCC (reverse); mt3: ATAAAAGAACCAATACGCCCTT (forward) and ATTAAGTCCTCCTCATGCTCCT (reverse); mt4: AAACCTATTCTTTTACACCCGACT (forward) and ATAGGACAATTCCGGTTAGACCAC (reverse); mt5: ATTCACACACCAAAAGGACGAAC (forward) and GAATGCAACCATGATCAACGCTA (reverse); mt6: CCCATATGAATGCGGATTTGACC (forward) and TTATAGTACGGCTGTGAATTCGTT (reverse); mt7: CATCAGCCCAAAACTAATTACAGG (forward) and TTTTCGGATGTCTTGTTCGT (reverse); mt8: TCCCCTAGTCTCCATTAACGA (forward) and AAGCCTCCTCAAATTCATTCGAC (reverse); mt9: AGGCTTCTCAGTAGACAAAGCTA (forward) and TAACAAGCATGAATAATTAGCCTT (reverse). The PCR product was purified using a PCR product purification kit (Shanghai Shenggong).

### Nuclear DNA (nDNA) preparation

According to the method described [31], the nuclei of liver cells were isolated and nDNA was then extracted from the nuclei by phenol-chloroform and was sheared by sonication (Soncis-VCX-130, Soncis) into small fragments prior to injection.

### Preparation of mitochondrial debris solution (MTD)

Isolated liver mitochondria were disrupted by sonication (Soncis-VCX-130) in PBS (1 g in 5 ml PBS) containing proteinase inhibitors (Roche) on ice. Centrifuge with 12,000 g at 4°C for 10 min. Transfer the supernatant (MTD) and store it at −80°C.

### Protein concentration measurement

Protein concentrations of various samples were determined by BCA kit according to the manual instruction (Beyotime Institute of Biotechnology, Shanghai).

### Plasma DNA measurement by Picogreen colorimetric assay

Blood samples were collected from mouse tails into tubes containing EDTA, and were centrifuged 3,000 g at 4 C for 10 min. Supernatant was transferred to a fresh tube and stored at −20 C. We used Quant-IT PicoGreen dsDNA Assay Kit [Invitrogen, USA] to measure DNA concentration in the plasma samples according to the manual instructions. Briefly, 1 μl of thawed plasma sample was mixed with 99 μl of TE buffer (10 mM Tris.HCL, 1 mM EDTA, pH7.5), followed by addition of 100 μl of Picogreen working solution. After incubation at room temperature for 3–5 min, the samples were measured by using SpectraMax M5 Multi-Mode Microplate Reader with 490 nm light for excitation. A standard curve was simultaneously prepared using Lambda DNA diluted at 1,000, 500, 250, 50, 10 and 0 ng/ml), respectively, as standard.

### Culture and treatment of immortalized human podocytes

Conditionally immortalized human podocyte cell line was from Dr. Moin Saleem at Bristol University, UK [32]. The methods described [33] were followed for podocyte culture and differentiation. The cells were treated with 50 μg/ml purimycin aminonucleoside (PAN, Sigma), 10 μg/ml lipopolysaccharide (LPS, Sigma), 20 μg/ml mtDNA, 20 μg/ml nDNA, and 100 μg/ml MTD protein, respectively, for 2 h. The cells were lysed and analyzed with immunoblotting.

### Immunoblotting

We followed the standard protocol for SDS-PAGE electrophoresis, membrane transferring, antibody incubation, and Chemluminescence development (ECL, Millipore) [33]. The antibodies used in this study, VDAC, phosphorylated p38 MAPK, and GAPDH were all purchased from Cell Signaling Technology (CA, USA).

### Treatment of mice with mtDAMPs

Balb/c mice were divided into four groups and treated as follow, 1) PBS group: injected with PBS; 2) mtDNA group: injected with 33.4 μg of mtDNA for a concentration in circulation (20 μg/ml) that was significantly higher than the clinical range (~ 5 ng/ml); 3) MTD group: injected with MTD containing 100 μg of protein which resulted in 60 μg/ml MTD protein in circulation, and 4) nDNA group: injected with 33.4 μg of nDNA for 20 μg/ml nDNA in circulation. In another experiment, Balb/c mice were either injected with PBS or 75 μg mtDNA, followed by urine collection. All injections were performed through tail vein of the animals.

### Treatment of rats with mtDAMPs

SD rats were divided into two groups, PBS group injected with 3 ml PBS, and MTD groups with 3 ml of MTD containing 7,600 μg of protein (equivalent to the amount of mitochondria from approximately 30% of a rat liver). Blood, urine, kidney and lung samples were collected for analysis at 3, 6, 12 or 24 h following injection.

### Measurement of albuminuria in mice

The urinary albumin and creatinine levels in the mice were measured using Albuwell M and Creatinine Companion Kits (Exocell Laboratories) according to the manufacturer’s instructions.

### Measurement of proteinuria in rats

Rat urinary protein was measured with Bradford method and urine creatinine was measured using HITACHI 7080 Biochemical auto-analyzer.

### Hematoxylin-eosin (H&E) and Periodic Acid-Schiff (PAS) staining

We performed H&E and PAS staining according to the methods described [34].

### Statistical analysis

Student's t-test was used to compare two groups; and one-way analysis of variance (ANOVA) and Dunnett's test were used for the comparison of multiple groups. Data are expressed as means±SE, and P-value <0.05 was considered significant.

## Results

### Preparation and characterization of mtDAMPs samples from mouse or rat liver

To test whether circulating mtDAMPs are capable of inducing proteinuria or kidney injury in mice or rats, we first prepared mtDAMPs samples, including mtDNA and mitochondrial debris solution (MTD) which contains all mtDAMPs, from mouse or rat liver, followed by quality assessment. We successfully obtained pure mitochondrial preparations as shown with microscopy (S1 Fig).

To prepare mtDNA, we chose to amplify mtDNA fragments by PCR instead of extracting intact mtDNA from purified mitochondria. This is because mtDNA amount is very low in a cell and only 0.5–1.0 μg intact mtDNA could be obtained from 1 g of liver (S2A Fig), making it difficult to prepare sufficient amount of mtDNA for animal treatment. In fact, PCR-generated mtDNA fragments have been used as cell injury inducers [6]. We then performed PCR to amplify 9 mtDNA fragments that cover the full-length mtDNA (S2B Fig). Additionally, we also amplified a mtDNA fragment corresponding to the region between no. 7767–8187 nt on mtDNA, which is 421 bp in length (S2C Fig) and contains 23 CpG motifs. This fragment has been demonstrated to be immune active [6].

We also isolated nuclei from liver cells (S2D Fig), and used them to prepare nDNA. The nDNA was sheared by sonication for the size ranging from 500–1,000 bp (S2C Fig). nDNA is known to have a much lower unmethylated CpG frequency [35] compared with mtDNA. nDNA was used as control in the study.

**Fig 1:**
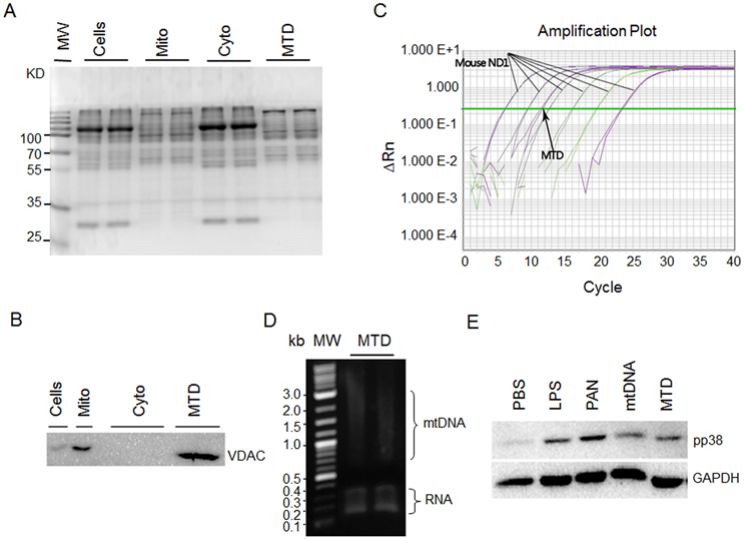
Preparation and characterization of mtDAMPs. **A.** SDS-PAGE and Coomassie staining of the samples indicated. Cells, whole cell lysates; Mito: whole mitochondrial lysates; Cyto: cytoplasmic fraction lysates; MTD: mitochondrial debris solution. **B.** Western blotting of VDAC in the samples indicated, showing the enrichment of the mitochondrial marker in MTD and mitochondrial lysates. **C.** qPCR analysis of the mtDNA in the MTD using the primers of mouse mitochondrial gene ND1 and serially diluted mtDNA plasmid samples for standard curve (see Methods). **D.** Agarose analysis of MTD, showing the presence of mtDNA, which was sheared in the sonication. **E.** Western blotting of phosphorylated p38 in the podocytes treated as indicated, demostrating that the mtDNA and MTD samples retained DAMP activity.

To assess the quality of MTD samples, we compared the proteins in the MTD, whole mitochondrial lysate, whole liver cell lysate, and liver cell cytoplasma fraction by SDS-PAGE and Coomassie blue staining. We found that the MTD exhibited a staining pattern that was similar to that of whole mitochondrial lysates (Fig 1A), suggesting that the majority of mitochondrial proteins are soluble and were included in the MTD sample. As expected, the staining pattern of the MTD was completely different from that of whole liver cells or their cytoplasmic fraction (Fig 1A), indicating that the proteins in MTD were mitochondrial specific. To further prove the purity of the MTD, we performed immunoblotting of VDAC, a mitochondrial specific protein. We found that VDAC was enriched in the MTD or whole mitochondrial lysate (Fig 1B), but was almost absent in the whole cell lysate or cytoplasmic fraction. To confirm that the MTD sample contained mtDNA, we performed both qPCR and agarose gel analyses, and mtDNA was detected in both assays (Fig 1C and 1D) with an expected abundance (data not shown).

To test whether the mtDAMP samples retain DAMP activity, we treated cultured human podocytes with the mtDNA, MTD, PAN or LPS (positive control), and examined their effects on p38 MAPK phosphorylation. p38 activation (phosphorylation) is one of the inflammatory pathways [8], and PAN and LPS are known to induce p38 phosphorylation. Immunoblotting showed that both the mtDNA and the MTD induced p38 phosphorylation in the cells (Fig 1E), indicating that these mtDAMP samples were functional and could be used in *in vivo* studies with mice or rats. This result also suggested that podocytes possess PRRs and their downstream signaling components, thereby responding to DAMPs. In fact, we have previously profiled the gene expression in the podocytes by microarray analysis and have detected the expression of a number of PRRs, including formyl peptide receptors and Toll-like receptors (S1 Table).

### Characterization of the mice treated with mitochondrial DAMPs

**Fig 2:**
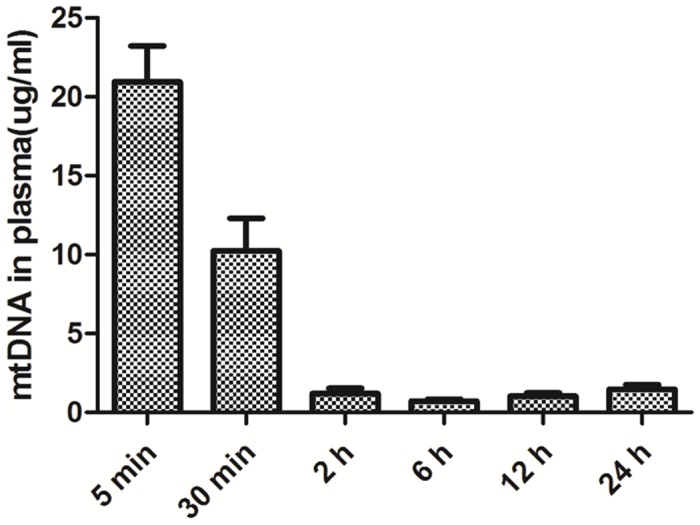
The kinetics of injected mtDNA in circulation. Picogreen colorimetric assay of the injected mtDNA in the plasma of mice (n = 3).

To determine whether mtDNA is capable of inducing proteinuria in mice, we decided to inject large amount of mtDNA to mice to achieve a concentration in circulation that is above the clinical range (5 ng/ml) [22, 24, 25]. To determine whether a concentration that is above the clinical range can be achieve by one injection, we injected 75 μg mtDNA into mice (n = 3) and collected plasma samples at 5 min, 30 min, 2 h, 6 h, 12 h and 24 h, respectively, following the injection. The plasma samples were also collected prior to mtDNA injection to determine the basal level of DNA in circulation. We used highly sensitive Picogreen colorimetric assay to measure the DNA concentration in the plasma samples. After subtracting the basal level value from each measurement, we obtained the kinetics of injected mtDNA in circulation (Fig 2), in which mtDNA concentrations decreased rapidly from ~ 21 μg/ml at 5 min to ~ 1 μg/ml at 2 h and thereafter. This indicated that one injection with 75 μg mtDNA is sufficient to achieve a concentration which is constantly well above the clinical range (5 ng/ml) throughout the 24 hours following the injection. The rapid decrease followed by stabilization of mtDNA concentration in circulation might be caused a rapid diffuse of mtDNA in the body, followed by equilibrium between plasma and tissues shortly.

**Fig 3:**
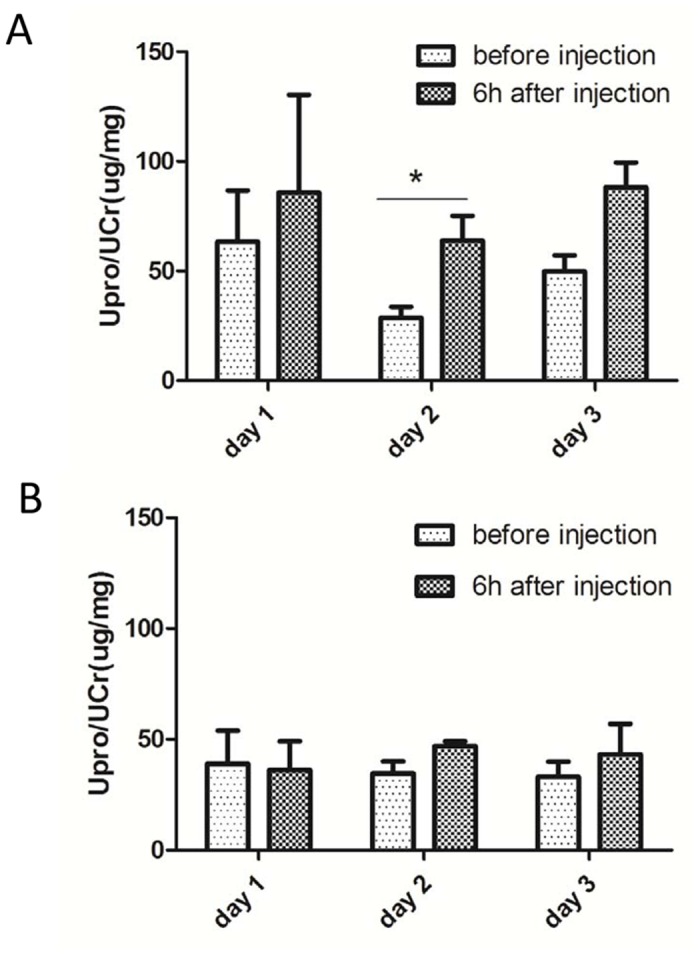
The effect of circulating mtDNA on urinary protein level in mice. **A.** Urinary albumin/creatinine levels in mice (n = 4) before and after the injection of 75 μg of mtDNA. The injection was performed on 3 consecutive days, and spot urine samples were collected 4–7 hours following each injection. *P < 0.05. **B.** Equal volume of PBS was injected into mice (n = 4) as control, and the spot urine samples were collected as assayed similarly as in “A”.

Based on the kinetics study of injected mtDNA in circulation, we injected 75 μg mtDNA (the mix of the 9 PCR products of mtDNA fragments) in PBS into mice (n = 4), and the equal volume of PBS (vehicle) into mice as control (n = 4). These mice received the same injections on 3 consecutive days. At each injection, spot urine samples were collected before and after the injection at 4 h to 7 h following the injection to ensure a successful urine collection from all mice. We found that urinary albumin levels always tended to elevate in the mice treated with mtDNA (Fig 3A) but not those with PBS (Fig 3B) after each injection. However, the increase of urinary albumin in the mice treated with mtDNA was subtle, and no high level of albuminuria was observed in any mice examined. In addition, histological examination of the kidneys of the mice sacrificed after the last injection revealed no any morphological abnormalities (data not shown).

We also tested the mtDNA fragment previously shown to be immune active, hoping that it would be more potent than the full-length mtDNA in inducing proteinuria. We first injected a smaller amount of this DNA fragment, i.e., 33.4 μg, for an initial concentration of ~ 20 μg/ml in circulation in mice. Urine and kidney samples were collected 1, 3, or 6 h following injection. We found neither proteinuria nor any other renal histological lesions in the mice (data not shown).

We next increased the dose and injected 75 μg of the mtDNA fragment to mice, which would result in an initial concentration of 45 μg/ml in circulation. Fifteen mice were treated with mtDNA and other 5 with PBS as control. Pooled urine samples from the 15 treated mice (3 pools, each with 5 mice) and the 5 control mice (1 pool with all 5 mice) were collected at 2 or 6 h following injection. Analysis with PAGE and silver staining revealed higher levels of albumin (100–150 μg/ml) in the urine of the mice treated with mtDNA compared with control mice (S3 Fig), however, these levels of urinary albumin were too low to be considered as albuminuria. As a control, nDNA was also tested in parallel and was found to exert no effect on urinary protein level in the mice (data not shown).

Next, we tested whether mitochondrial debris solution (MTD) is effective in inducing proteinuria in mice. We injected 100 μg MTD proteins for an initial concentration of ~ 60 μg/ml in circulation. However, we found neither increases in urinary protein nor renal histological lesions in the mice (data not shown). Nevertheless, subtle widening of alveolar interstitium and mononuclear cells infiltration were occasionally observed in the lung of the mice (S4 Fig), indicating that systemic inflammation was induced by MTD in the mice.

### Characterization of rats treated with mitochondrial DAMPs

Next we used SD rats to further examine the effects of MTD on urinary protein. Since the clinical range of plasma mtDNA in trauma patients is ~ 5 ng/ml, and the ratio of mtDNA versus mitochondrial protein in weight is approximately 1:5,000 [36], we estimated that the concentration of accompanied mitochondrial proteins in the patients should be about 25 μg/ml (5× 5,000 ng/ml). Therefore, we injected MTD containing 2,000 μg of proteins (equivalent to the mitochondria from 5–10% of the liver of a rat) into the rats for a concentration of ~ 100 μg/ml in circulation. This level should be well above the clinical range. However, we did not observe proteinuria or other renal lesions in the rats (data not shown).

**Fig 4:**
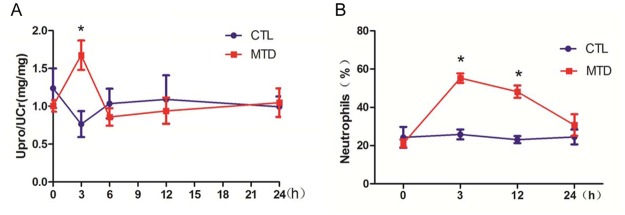
The effect of injected MTD on urinary protein level and neutrophils percentage in rats. Rats were treated either with PBS (CTL, n = 5) or MTD (7,600 μg/rat) (n = 12). **A.** The ratios of urinary protein versus creatinine at different time points. *P < 0.05, denoting a difference that was statistically significant. Upro: urinary protein; Ucr: urinary creatinine. **B.** Inflammation examination by the percentages of neutrophils in the blood of the MTD-treated rats (n = 6) and PBS-treated control rats (n = 4). *P < 0.05.

**Fig 5:**
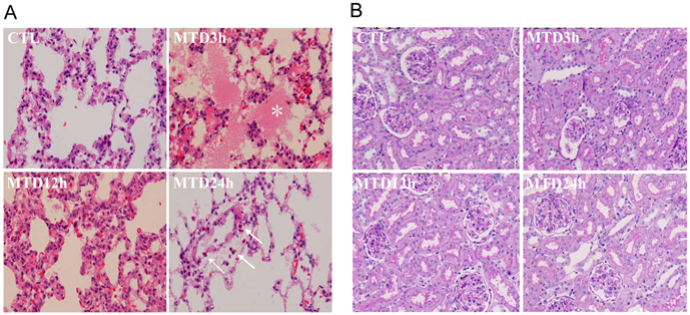
Histological analysis of the lungs and kidneys of the rats treated with MTD or PBS (CTL). **A.** H & E staining of the lungs of the rats, showing the presence of exudates in some alveoli (indicated by white *) at the 3 h following MTD injection. Infiltrated neutrophiles were observed in the lung interstitium (white arrows). **B.** PAS staining of the kidneys of the rats, showing that the kidneys of all rats were grossly normal in morphology.

We then injected 7,600 μg of MTD proteins (equivalent to the mitochondria from ~ 30% of the liver of a rat) to the rats, resulting in an initial concentration of 450 μg/ml in circulation, which was much higher than the clinical range (18 folds). We analyzed the spot urine samples from the rats at 3, 6, 12, or 24 h following the injection and found an increase in urinary protein at 3 h but not 6 h and thereafter (Fig 4A). The rats also had increased percentages of neutrophils in the blood, which peaked at 3 h following the treatment, and gradually returned to normal level at 24 h (Fig 4B). Meanwhile exudates were seen in the alveoli of the lung at 3 h following the injection, which was diminishing at 12 h and disappeared completely at 24 h (Fig 5A). Infiltration of immune cells was also observed in the alveolar interstitium (Fig 5A). These observations indicated acute systemic inflammation in the rats with the peak at 3h following MTD injection. Thus, it appeared that mild proteinuria was transiently present only in the peak phase of inflammation. On the other hand, the kidney histology of the treated rats was normal (Fig 5B) and the serum creatinine levels of the rats were also normal (S5 Fig).

**Fig 6:**
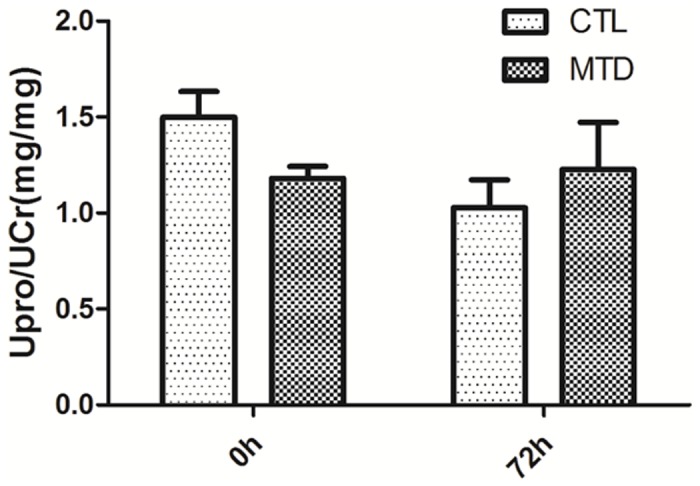
The effect of multiple injections of MTD on urinary protein levels in rats. 7,600 μg of MTD was injected into rats (n = 6) and an equal volume of PBS was injected to another group of rats (n = 4) on 3 consecutive days. Spot urine samples were collected before the first injection and 24 h after the last injection (i.e., 72 h since the first injection). Urinary protein levels were assayed and no significant difference was found between the two groups of rats in urinary protein level after the treatment of 3 injections.

Lastly, we performed multiple injections of MTD into rats, hoping that it could eventually lead to more severe proteinuria in the end. We injected MTD proteins (7,600 μg) into each rat on 3 consecutive days, and collected spot urine samples 24 h following the last injection, i.e., 72 h since the first injection. Urinary protein and creatinine assays showed that there was no significant difference in urinary protein level between the rats treated with MTD and PBS (control) (Fig 6). Furthermore, the kidneys of MTD-treated rats were normal morphologically (data not shown), and their inflammation was not aggravated as shown by neutrophil counting and lung lesion examination (data not shown).

## Discussion

Due to the findings of recent studies that mtDNA and formyl peptides are DAMPs and are capable of inducing cytokines, inflammation and SIRS [6, 8, 27], that cytokines can induce renal cell injury leading to proteinuria, that renal intrinsic cells express a variety of PRRs, and that mtDAMPs can directly increase endothelial cell permeability *in vitro*, we hypothesize that the increased circulating mtDAMPs are responsible for the proteinuria in the patients with trauma or burn injury. We therefore investigated the effect of circulating mtDAMPs on kidney. However, neither mtDNA nor MTD was capable of inducing detectable proteinuria or renal injury when they were injected into rodents with a dose resulting in a concentration within or above the clinical range. Therefore, we conclude that the circulating mtDAMPs in trauma or burn patients may not be responsible for the observed proteinuria, thus ruling out mtDAMPs as circulating factors that cause proteinuria and kidney injury. Our study may have also excluded them as therapeutic targets for the treatment of proteinuria in trauma or burn patients.

The limited effect of mtDNA on proteinuria development as observed in the present study may be consistent with the study by Ries et al in which mtDNA from platelets was unable to induce IFN-alpha [37], which is routinely induced by CpG ODN 6016, suggesting that mtDNA may not be as potent as CpG ODN in inducing cytokine production in term of both induction efficiency and cytokine spectrum. Crouser et al also found that mtDNA is not effective in inducing IL-8 production in monocytes [38]. These studies have raised a possibility that mtDNA might have become adapted to the hosts and thus confers a reduced DAMP activity. In fact, the CpG frequency of mtDNA has become lower than that of bacterial DNA [35]; and the CpG methylation degree in mtDNA has been found higher than the previously estimated 3–5% [39]. Lowered CpG frequency and increased methylation might have led to a reduction of mtDNA potency as a DAMP molecule. Moreover, since the potency of CpG motifs as a ligand is also dependent on the sequences surrounding CpG motifs [40], mtDNA might have changed the surrounding sequences to reduce its DAMP activity, leading to a better survival of mitochondria (the ancient bacteria) in the hosts. An additional reason for the failure of mtDNA in inducing proteinuria is that, glomerular cells do not express TLR9, the receptor for unmethylated CpG ligand, in normal rodents (our unpublished observation, data not shown), therefore circulating mtDNA is unlikely to act directly on glomerular cells to cause injury and proteinuria.

The injection of MTD, which contains all mtDAMPs including mtDNA, also failed in inducing proteinuria although MTD is supposed to be more potent than mtDNA. Only an extremely high dose of MTD gave rise to subtle urinary protein increase transiently at the peak phase of acute systemic inflammation (which was shown by the neutrophil percentage increase and lung lesions at 3h following MTD injection). Since the resulting concentration of mtDAMPs in circulation by MTD injection does not exist in patients, this experiment is clinically irrelevant. Therefore, we conclude that circulating mtDAMPs with a concentration in the clinical range have no effect on kidney.

However, one could argue that different species or the same species with differential genetic background could have different sensitivities to circulating mtDAMPs, therefore our conclusion that circulating mtDAMPs in clinical range cannot induce proteinuria may not apply to trauma or burn patients because human could have a much higher sensitivity to mtDAMPs than rodents. This possibility is low but still warrants further investigation.

Other explanations of the failure of mtDAMPs in inducing proteinuria or kidney injury could be 1) that other events, eg., haemorrhage, sepsis, or glomerular endothelial disruption, may be required as synergistic factors for the effect of mtDAMPs on proteinuria induction, but these events apparently lacked in the models used in this study; 2) that the cytokines or chemokines induced by circulating mtDAMPs do not include those that are capable of damaging renal cells; and 3) that circulating mtDAMPs alone are not sufficient to damage renal intrinsic cells and require other types of DAMPs or factors to achieve renal cell injury. Particularly for the PCR-generated mtDNA, its potency might be impaired by lack of the proteins that are bound to it in cells normally, including Tfam which is capable of inducing immune response [41, 42]. The idea that synergistic actions of mitochondrial molecules are required for their effects has been demonstrated by a recent study, in which Sun S and colleagures showed that mtDNA or formyl peptides alone do not change (increase) the permeability of endothelial cells but MTD (entire mitochondrial debris solution) does [17]. It is necessary to investigate whether mtDAMPs could facilitate proteinuria and kidney injury through collaborating with other factors, eg., non-mitochondrial factors. In fact, non-mitochondrial factors are also released to circulation from the injured or dead cells in the patients.

Although the mtDAMPs in circulation have no damaging effects on kidney as demonstrated by the present study, it is possible that local mtDAMPs that are released by injured or dead cells in kidney can have impact on adjacent healthy cells because of the expression of many DAMPs receptors in the cells (S1 Table), thereby propagating or facilitating the renal injury. This issue warrants further investigation because it would possibly reveal a novel mechanism underlying the progression of renal disease.

## Supporting Information

S1 Fig The morphology of mouse and rat mitochondria prepared in this study under a light microscope. (TIF)

S2 Fig Preparation of mtDNA and nDNA. **A**. Agarose gel analysis of the intact mtDNA which was confirmed by a diagnostic digestion with Pci *I* restriction enzyme. **B**. Amplification of 9 mtDNA fragments (mt1 through mt9) that cover the full-length mtDNA. **C**. PCR-amplified mtDNA product (421 bp) and nDNA fragmented by sonication or not. Mt PCR: the PCR product of mitochondrial fragment for injection. frag.: fragmented by sonication; unfrag: not fragmented. **D**. Toluidine blue staining of the nuclei purified from mouse liver.
(TIF)

S3 Fig PAGE and silver staining of urine samples of the mice injected with overdose of mtDNA. Each mouse was injected with 75 μg mtDNA or saline (NS). Each lane represented the pooled urine sample from 5 mice (either control mice or the mice treated with mtDNA). Increased urine albuminu levels were shown in the three pooled urine samples at 6 h following injection. 20 μl of urine from each sample was loaded onto the gel. Note that one pooled urine sample of mtDNA treated mice at 2 h following injection was lost. The current image was assembled from the relevant lanes which were originally separated in a large gel.
(TIF)

S4 Fig The effect of mtDAMPs on lung. H & E staining of the lung tissues of the mice treated with PBS (CTL), mtDNA, MTD or nDNA as indicated. Subtle widening of alveolar interstitium and mononuclear cells infiltration were observed in the mtDNA or MTD-treated mice.
(TIF)

S5 Fig The effect of MTD on serum creatinine level of rats. The rats described in Fig 5, which were treated with MTD (n = 9) or PBS (n = 8), exhibited no difference in serum creatinine level.
(TIF)

S1 Table PRRs detectable in human podocytes. (DOC)
