# Supplementary figures and images for: Circulating Mitochondrial DAMPs Are Not Effective Inducers of Proteinuria and Kidney Injury in Rodents
Source: PLoS One. 2015 Apr 22;10(4):e0124469. doi: 10.1371/journal.pone.0124469 (PMC4406729; doi:10.1371/journal.pone.0124469)

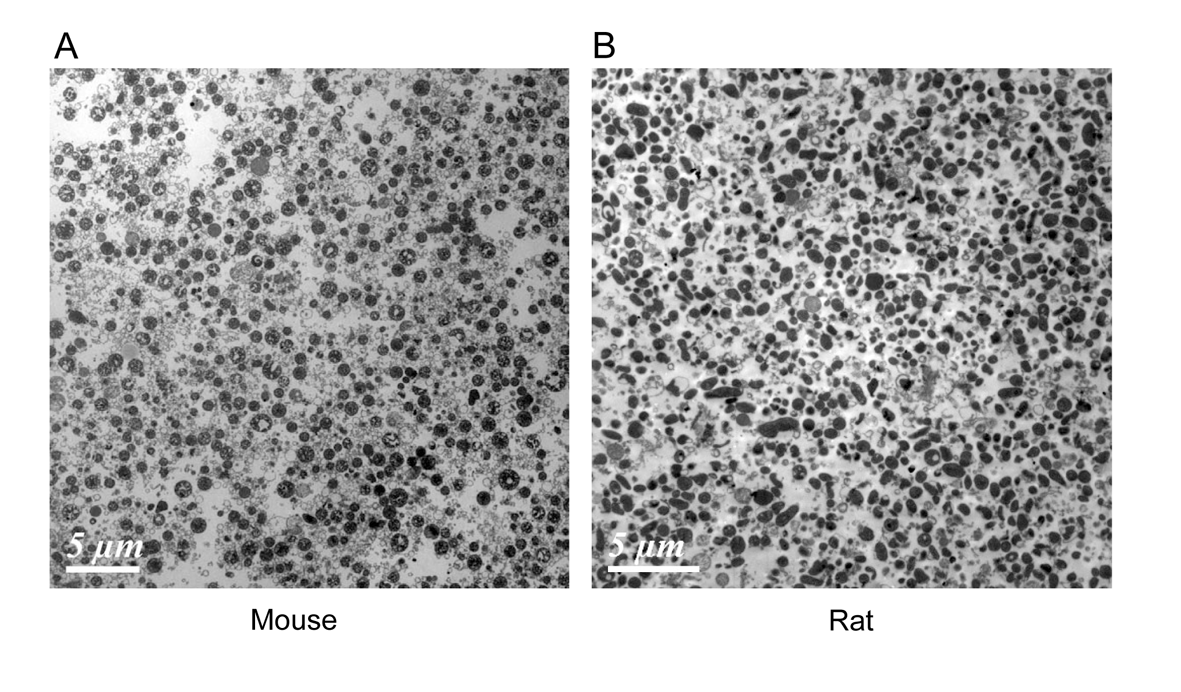

Supplement: S1 Fig — (TIF) [file pone.0124469.s001.tif]

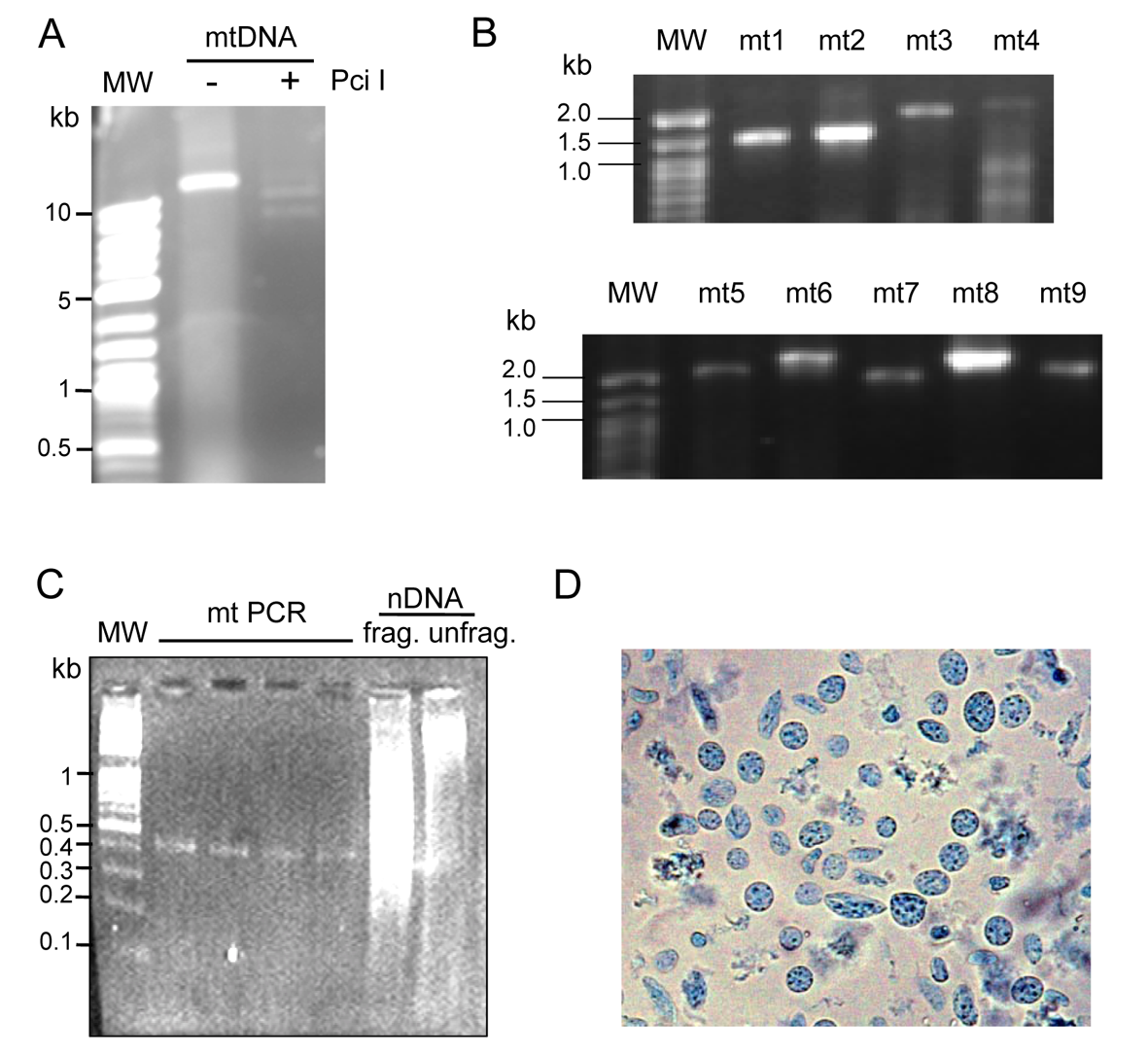

Supplement: S2 Fig — A. Agarose gel analysis of the intact mtDNA which was confirmed by a diagnostic digestion with Pci I restriction enzyme. B. Amplification of 9 mtDNA fragments (mt1 through mt9) that cover the full-length mtDNA. C. PCR-amplified mtDNA product (421 bp) and nDNA fragmented by sonication or not. Mt PCR: the PCR product of mitochondrial fragment for injection. frag.: fragmented by sonication; unfrag: not fragmented. D. Toluidine blue staining of the nuclei purified from mouse liver. (TIF) [file pone.0124469.s002.tif]

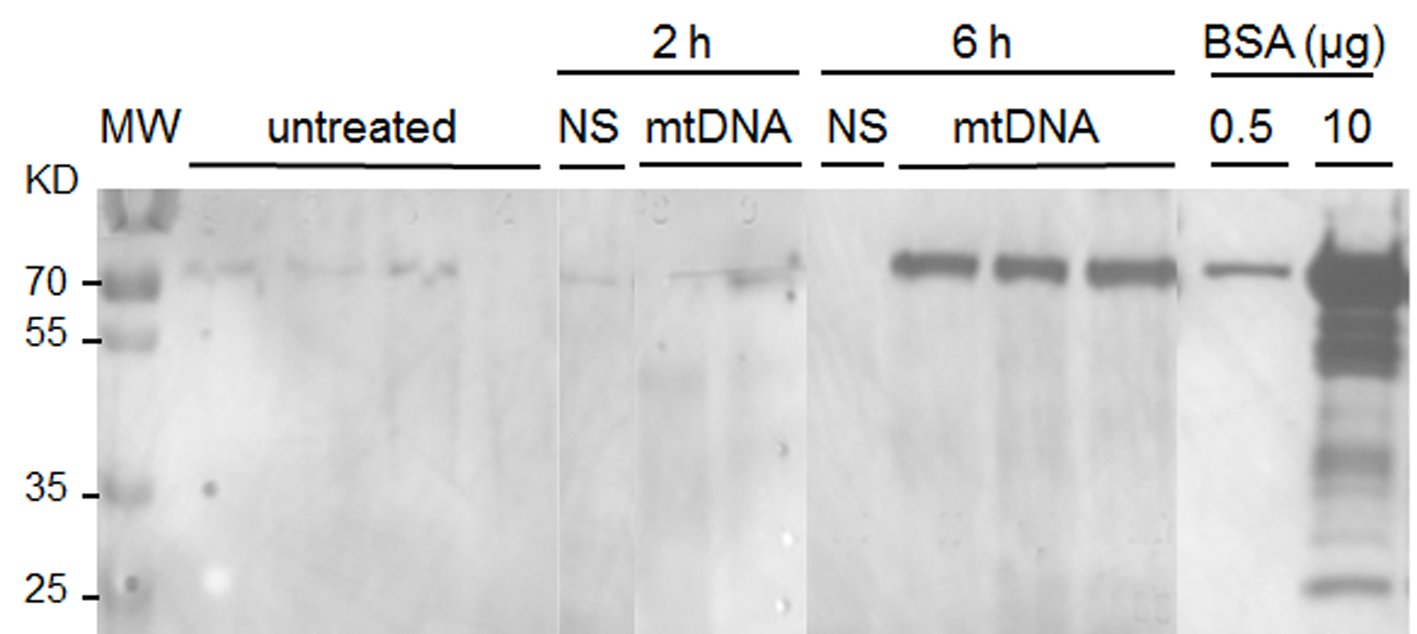

Supplement: S3 Fig — Each mouse was injected with 75 μg mtDNA or saline (NS). Each lane represented the pooled urine sample from 5 mice (either control mice or the mice treated with mtDNA). Increased urine albuminu levels were shown in the three pooled urine samples at 6 h following injection. 20 μl of urine from each sample was loaded onto the gel. Note that one pooled urine sample of mtDNA treated mice at 2 h following injection was lost. The current image was assembled from the relevant lanes which were originally separated in a large gel. (TIF) [file pone.0124469.s003.tif]

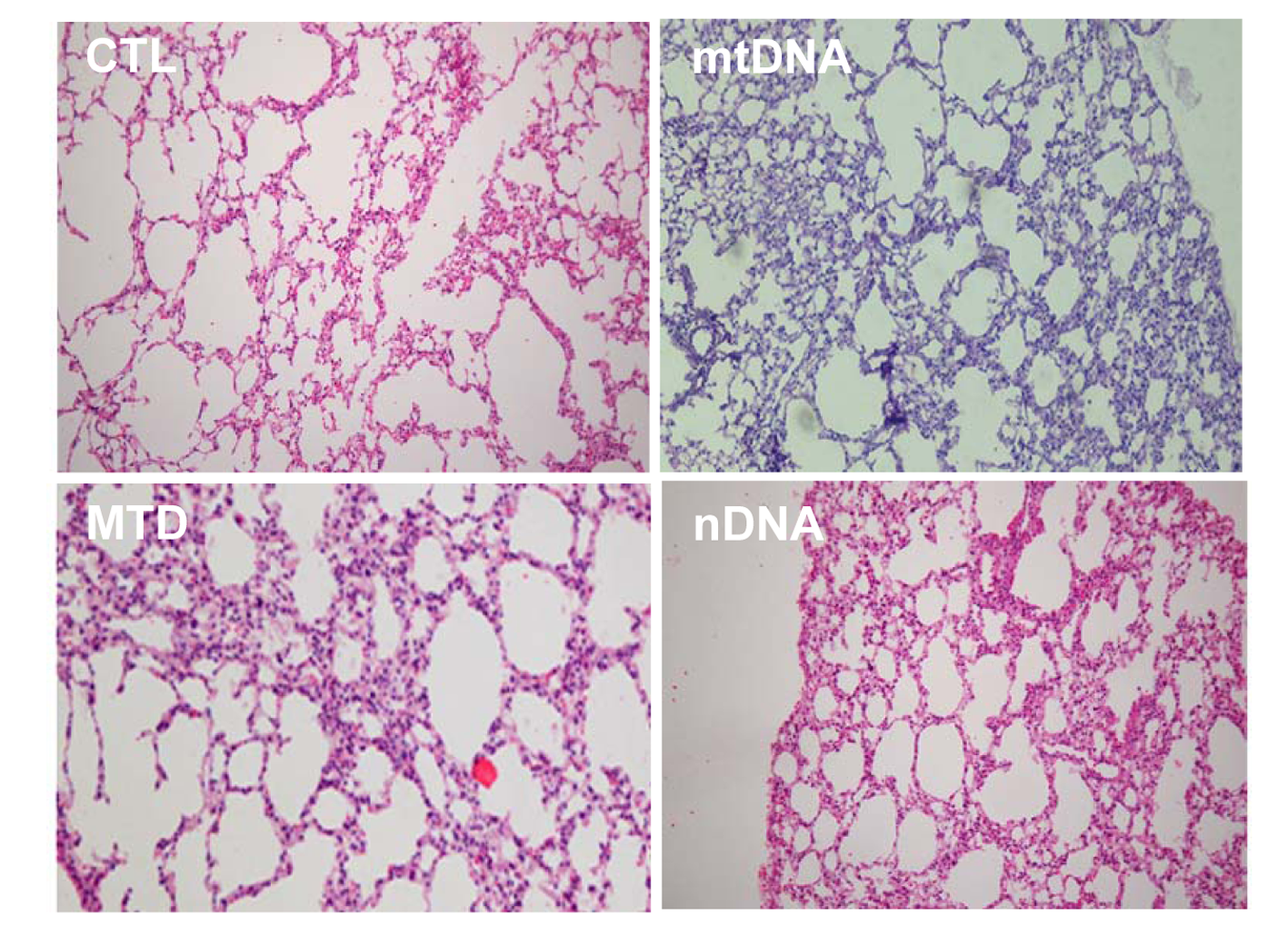

Supplement: S4 Fig — H & E staining of the lung tissues of the mice treated with PBS (CTL), mtDNA, MTD or nDNA as indicated. Subtle widening of alveolar interstitium and mononuclear cells infiltration were observed in the mtDNA or MTD-treated mice. (TIF) [file pone.0124469.s004.tif]

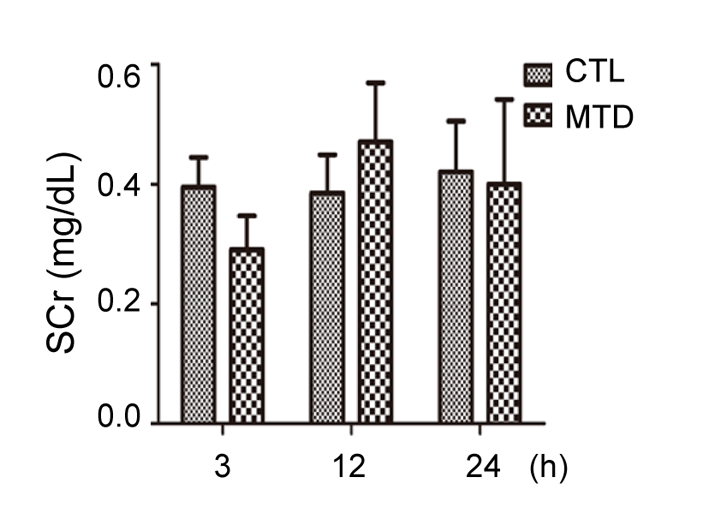

Supplement: S5 Fig — The rats described in Fig 5, which were treated with MTD (n = 9) or PBS (n = 8), exhibited no difference in serum creatinine level. (TIF) [file pone.0124469.s005.tif]
