# Supplementary material for: Circulating Mitochondrial DAMPs Are Not Effective Inducers of Proteinuria and Kidney Injury in Rodents
Source: PLoS One. 2015 Apr 22;10(4):e0124469. doi: 10.1371/journal.pone.0124469 (PMC4406729; doi:10.1371/journal.pone.0124469)
Supplement: S1 Table — (DOC) [file pone.0124469.s006.doc]

**Table S1**. **PRRs detectable in human podocytes**

| TLR1 | FPR1 | NLRP4 | CLEC10A | DDX58 |
| --- | --- | --- | --- | --- |
| TLR2 | FPR2 | NLRP5 | CLEC12B | NAIP |
| TLR3 | FPR3 | NLRP9 | MRC2 | IFIH1 |
| TLR4 | NLRP1 | NLRP14 | NOD1 | NLRC5 |
| TLR5 | NLRP2 | CLEC4E | RAGE | CIITA |
| TLR9 | NLRP3 | CLEC5A | NLRX1 |  |
